# HPS1 Regulates the Maturation of Large Dense Core Vesicles and Lysozyme Secretion in Paneth Cells

**DOI:** 10.3389/fimmu.2020.560110

**Published:** 2020-11-05

**Authors:** Jiaying Yu, Xin He, Aihua Wei, Teng Liu, Qin Zhang, Ying Pan, Zhenhua Hao, Lin Yang, Yefeng Yuan, Zhao Zhang, Chang Zhang, Chanjuan Hao, Zhihua Liu, Wei Li

**Affiliations:** ^1^ Beijing Key Laboratory for Genetics of Birth Defects, Beijing Pediatric Research Institute, MOE Key Laboratory of Major Diseases in Children, Genetics and Birth Defects Control Center, National Center for Children’s Health, Beijing Children’s Hospital, Capital Medical University, Beijing, China; ^2^ University of Chinese Academy of Sciences, Institute of Genetics and Developmental Biology, Chinese Academy of Sciences, Beijing, China; ^3^ Department of Dermatology, Beijing Tongren Hospital, Capital Medical University, Beijing, China; ^4^ Institute for Immunology, Tsinghua-Peking Center for Life Sciences, School of Medicine, Tsinghua University, Beijing, China

**Keywords:** Hermansky-Pudlak syndrome, HPS1, large dense core vesicle, Paneth cell, inflammatory bowel disease, VAMP7

## Abstract

HPS1, a BLOC-3 subunit that acts as a guanine nucleotide exchange factor of Rab32/38, may play a role in the removal of VAMP7 during the maturation of large dense core vesicles of Paneth cells. Loss of HPS1 impairs lysozyme secretion and alters the composition of intestinal microbiota, which may explain the susceptibility of HPS-associated inflammatory bowel disease. Hermansky-Pudlak syndrome (HPS) is characterized by oculocutaneous albinism, bleeding tendency, and other chronic organ lesions due to defects in tissue-specific lysosome-related organelles (LROs). For some HPS subtypes, such as HPS-1, it is common to have symptoms of HPS-associated inflammatory bowel disease (IBD). However, its underlying mechanism is largely unknown. HPS1 is a subunit of the BLOC-3 complex which functions in the biogenesis of LROs. Large dense core vesicles (LDCVs) in Paneth cells of the intestine are a type of LROs. We here first report the abnormal LDCV morphology (increased number and enlarged size) in HPS1-deficient *pale ear* (*ep*) mice. Similar to its role in melanosome maturation, HPS1 plays an important function in the removal of VAMP7 from LDCVs to promote the maturation of LDCVs. The immature LDCVs in *ep* mice are defective in regulated secretion of lysozyme, a key anti-microbial peptide in the intestine. We observed changes in the composition of intestinal microbiota in both HPS-1 patients and *ep* mice. These findings provide insights into the underlying mechanism of HPS-associated IBD development, which may be implicated in possible therapeutic intervention of this devastating condition.

## Introduction

Hermansky-Pudlak Syndrome (HPS) is a recessive rare genetic disease. So far, 11 HPS subtypes (HPS-1~HPS-11) have been identified. The protein complexes associated with these HPS genes are named as HPS protein associated complexes (HPACs), including AP-3 (Adapter Protein-3), BLOC (biogenesis of lysosome-related organelles complex)-1, BLOC-2, BLOC-3, and HOPS (homotypic fusion and protein sorting complex) (1). HPACs have been known to function in the maturation and secretion of lysosome-related organelles (LROs). The main clinical features of HPS are oculocutaneous albinism, prolonged bleeding, and pulmonary fibrosis or inflammatory bowel disease (IBD) in some cases, which threaten the life of HPS patients (1). There are two major types of IBD, Crohn’s disease (CD) and ulcerative colitis. HPS patients with IBD mostly have symptoms at adolescence with characteristics of CD (2). CD symptoms have been reported in some cases of HPS-1, HPS-4, and HPS-6 subtypes (3, 4).

CD is thought to be a complex disorder associated with both genetic and environmental factors, characterized by abdominal cramps, fever, weight loss, malnutrition, frequent diarrhea, and blood in the stool. Individuals carrying IBD susceptibility genes are more liable to environmental influences that may induce intestinal immune imbalance leading to inflammation (5). Twenty to 50% of patients with CD show abnormalities in the morphology and function of Paneth cells (PCs) (6). Paneth cells, located at the bottom of the small intestinal crypts, are highly specialized polarized cells with a large amount of mature large dense core vesicles (LDCVs) at the apical microvillar terminus. The number of PCs gradually increases from the end of the duodenum to the end of the cecum, with the most abundant distribution at the end of the small intestine (7). A number of CD susceptibility genes are associated with the function of PCs. Previous studies have proposed the concept of “Paneth disease” to describe a series of intestinal diseases caused by abnormal PCs, suggesting association between PC dysfunction and defective antibacterial ability in CD (8). PCs execute immune function through the degranulation of LDCVs containing lysozyme, defensins (also known as cryptdins in mice), secreted phospholipase A2, C-type lectin Reg3γ and other anti-microbial peptides (AMPs) (9).

LDCV is a type of LROs (1, 10, 11) which mainly present in nerve cells, exocrine cells, and endocrine cells (12–14), to store and secrete contained molecules (15). LDCV originates from trans-Golgi network (TGN) (16). Within PCs, various AMPs are selectively sorted and transported into LDCVs after their synthesis from TGN. Sorting of lysozyme, a major component of PC LDCVs, is directed by commensal bacteria through the NOD2-LRRK2-Rab2a axis. Upon microbial sensing, NOD2 recruits LRRK2, and subsequently Rab2a, on LDCVs, which retains lysozyme in mature LDCVs, Loss of NOD2, LRRK2, or Rab2a from the surface of LDCVs results in lysosomal degradation of lysozyme in PCs (17). However, the regulatory mechanisms for the biogenesis and secretion of PC LDCVs are largely unknown.

HPS-1 is the predominant subtype accounting for 47.5% of reported HPS cases in the Chinese population (18). HPS1 and HPS4 proteins are found in the BLOC-3 complex. The known function of BLOC-3 is to recycle v-SNAREs on stage IV melanosomes (19) by acting as a GDP/GTP exchange factor (GEF) of Rab38 or Rab32 (20). Dysfunction of BLOC-3 leads to the retention of VAMP7 in melanosomes that affects their maturation (19) and exocytosis (21). Accumulating case reports have shown that among 11 HPS subtypes the incidence of HPS-1 is much higher than the others, and at least 10% of the reported HPS-1 cases had colitis or CD-like IBD (3). However, the role of HPS1 deficiency in the pathogenesis of CD-like IBD is still unclear. BLOC-3 is ubiquitously expressed and PC LDCV has LRO features like melanosome. However, whether HPS1 or BLOC-3 plays a role in the function of PC LDCVs, which is likely associated with the CD-like manifestations, remains elusive.

Here we use the HPS1-deficient mouse model, *pale ear* (*ep*), to explore the underlying mechanism of HPS-associated IBD. For the first time, we report compositional changes in the fecal microbiome of HPS-1 patients and *ep* mice and the abnormal secretion of lysozyme in *ep* PC LDCVs. This establishes a link between the dysfunction of PC LDCVs and the susceptibility of HPS-associated IBD.

## Material and Methods

### Mice

The HPS1 deficient *ep* mice and C57BL/6J mice (WT) were originally obtained from Jackson Laboratory, and maintained at Dr. Richard T. Swank’s lab. Mice were bred under specific pathogen-free conditions in the animal facility of the Institute of Genetics and Developmental Biology (IGDB), Chinese Academy of Sciences. Genotyping primers for the *ep* mutant are 5’-ACTGTGGGGTGGACATTTGG-3’ and 5’-AGTGATGCGCCCTAGGCAAT-3 (348 bp amplicon), and primers for WT *Hps1* are 5’-ACTGTGGGGTGGACATTTGG-3’ and 5’-AGAAGCCTGCAAGCAAGACG-3’ (264 bp amplicon). Age and gender matched WT and *ep*/+ mice were used as controls. All animal experiments were approved by the Institutional Animal Care and Use Committee of IGDB.

### Patients

Patients recruited in this study were diagnosed with HPS-1 at Beijing Tongren Hospital from 2013 to 2018. Diagnosis of HPS was based on clinical manifestations, absence of platelet dense granules under electron microscopy, loss of platelet HPS1 or HPS4 protein by Western blotting (18). Mutations of the *HPS1* gene confirmed the diagnosis of HPS-1 by next-generation sequencing (22). Patient H006 was reported previously (22). Patient H014 and H015 were reported recently (18) (**Table S1**). Healthy controls are volunteers recruited from Beijing Tongren Hospital, Capital Medical University. Fecal samples of control and HPS1 patient families were obtained after written informed consents. Fecal sample and patient data used in this study were approved by the internal review board of the bioethics committees of Beijing Tongren Hospital, Capital Medical University. The study was conducted according to the declaration of Helsinki principles.

### Antibodies

The polyclonal HPS1 antibody was generated in New Zealand white rabbits against a His-tagged fusion protein corresponding to the mouse full-length *Hps1* gene (NM_019424). The polyclonal rabbit anti-procryptdin antibody was from Dr. Zhihua Liu’s Laboratory, Institute of Biophysics, Chinese Academy of Sciences. The FITC conjunct *Ulex europaeus* lectin (L9006) and monoclonal anti-β-Actin antibody (A5441) were purchased from Sigma. Rhodamine-labeled UEA-I (RL-1062) was purchased from Vector Laboratories. Anti-LYZ (C-term) rabbit polyclonal antibody (617544) and Rab2a rabbit polyclonal antibody (510764) were purchased from ZENBIO. Purified mouse anti-GM130 antibody (610822) and mouse anti-human LAMP-1 antibody (611043) were obtained from BD Biosciences. TfR antibody (OX26) (NB200-585SS) was purchased from Novus Biology. Rab38 antibody (9688) was purchased from Cell Signaling Technology. Rab32 antibody (SAB4200086) was purchased from Sigma-Aldrich (Darmstadt, Germany). VAMP7 monoclonal mouse purified IgG (232011) was purchased from Synaptic Systems (Goettingen, Germany). TGN38 (21-G) antibody (sc-101273) was purchased from SANTA CRUZ. HRP-labeled goat anti-Mouse IgG (ZB-2301) and HRP-labeled goat anti-Rabbit IgG (ZB-2305) were purchased from Zsbio, China. Alexa Fluor 488 Donky-anti-Rabbit IgG (A21206) and Alexa Fluor 488 Donky-anti-Mouse IgG (A28175) were purchased from Invitrogen.

### 16S rDNA Extraction and Microbiota Analysis

Sample collection, shipping, and DNA extraction were performed following the manufacturer’s protocols. Fresh stool sample were collected in collection tubes with stool DNA stabilizer (1038111300, Stratec, Germany) and 16S rDNA was extracted with PSP Spin Stool DNA Plus Kit (1038110300, Stratec, Germany). All extracted DNA samples were kept at -80°C before further analysis. The variable V4 region of 16S rRNA gene was amplified from samples by IonS5™XL platform using the primers 515F and 806R for bacterial diversity analysis. Cutadapt (V1.9.1, http://cutadapt.readthedocs.io/en/stable/) and Barcode were used to get the Raw Reads. The chimerical sequences were removed from the Raw Reads by comparing reads sequences to the species detection chimeric sequences (https://github.com/torognes/vsearch/) (23) to get the Clean Reads. Uparse (v7.0.1001, http://www.drive5.com/uparse/) were used for the clustering of the Clean Reads. Sequences with 97% of consistency (Identity) were clustered as the Operational Taxonomic Units (OTUs). Taxonomic analysis and community diversity were analyzed by the R software (Version 2.15.3) (24). Alpha diversity index analysis was carried out for different groups under 97% consistency threshold; the data volume cutoff = 60481 was selected during homogenization. T-test and Wilcox rank sum test were used for parametric test and non-parametric test, respectively. T-test was conducted between groups to find inter-group differences at different levels of classification (Phylum, Class, Order, Family, Genus, Species) with significant differences (*p* < 0.05). By default, the results of the gate level are displayed, and if there is no significant difference in the gate level, the next level is displayed. LDA Effect Size (25) analysis was performed by the LEfSe software (http://huttenhower.sph.harvard.edu/galaxy), the default filter value LDA Score = 4.4.

### Quantitative RT-PCR

Total RNA was extracted from distal ileum tissue of 5 pairs of age-matched WT and *ep* mice using RNeasy Mini kit (74104, Qiagen) and first-strand cDNA was generated with the iScript cDNA Synthesis Kit (1708890, Bio-Rad) according to the manufacturer’s protocols. qRT-PCR analysis was performed using SYBR PrimeScript Ready Mix (Takara) in an ABI 7900 sequence detection system (Applied Biosystems). GAPDH expression was used for normalization. Total RNAs are from the dissected trunk region of zebrafish embryos. The PCR primers are:

Gapdh-F: TCATCAACGGGAAGCCCATCAC,
Gapdh-R: AGACTCCACGACATACTCAGCACCG,
Lyz1-F: GCCAAGGTCTACAATCGTTGTGAGTTG,
Lyz1-R: CAGTCAGCCAGCTTGACACCACG,
Defcr1-F: TCAAGAGGCTGCAAAGGAAGAGAAC,
Defcr1-R: TGGTCTCCATGTTCAGCGACAGC.

### Immunoblotting

Mouse small intestine tissues were obtained from distal ileum and homogenized by RIPA lysis with protease inhibitor cocktail (Sigma-Aldrich). After 30 min incubation on ice, lysate was centrifuged at 12,000 g for 15 min to remove nuclei and cell debris. 20 μg of total protein per lane was loaded and separated within SDS-PAGE gels, and transferred to polyvinylidene difluoride membranes (PVDF, Millipore). The membrane was probed by antibodies described above and visualized with SuperSignal West Pico Plus (Thermo Fisher) by chemiluminescence imager (Sage Creation, China).

### Transmission Electron Microscope Assay

Mouse distal ileum sections were fixed over night by 0.1 M sodium phosphate buffer (pH 7.2) containing 2.5% glutaraldehyde and 2% paraformaldehyde at 4°C and washed by double distilled water for 4 times to remove glutaraldehyde. Tissue sections were then fixed by 1% osmic acid for 1 h at 4°C followed by dehydration with an acetone gradient (30-50-70-80-90-100-100-100%). Infiltration and embedment with an Embed 812 kit (EMS #14120, Electron Microscopy Sciences) were operated as the protocol described. 70 nm slices were prepared with Leica EM UC7 Ultramicrotome and images were obtained with a JEM 1400 electron microscope (JEOL, Japan).

### Immunofluorescence Confocal Imaging

Mouse distal ileum tissue were fixed overnight with 4% paraformaldehyde in PBS buffer at 4°C and embedded with paraffin. 5 μm slices were prepared with a rotary paraffin microtome (Leica RM2255) and mounted on positively charged slides, followed by antigen retrieval of 20 min steaming with 0.01 M sodium citrate buffer (pH 6.0). Tissue slices were then blocked with 1% normal goat serum (AR0009, Wuhan booster, China) in PBS buffer for 30 min at room temperature, incubated in primary antibody solutions (lysozyme 1:200, procryptdin 1:50) at 4°C overnight, washed by PBS buffer for 3 times and incubated in secondary antibody solutions (1:1,000) and fluorescence conjunct lectin. Nucleus were stained by DAPI while mounting. Confocal images were acquired with a 100xoil objective with NA 1.40 on an ECLIPSE Ti-C2 confocal microscope (Nikon, Japan). Images were obtained using the NIS-Elements AR 3.2 software provided by Nikon, and analyzed with NIH Image J.

### Image Analysis

Quantification of LDCVs and Paneth cell phenotype was done by two blinded investigators independently. Five randomly selected images acquired by a 100× oil objective with NA 1.40 per animal were quantified. For degranulation analysis of hematoxylin-eosin (H-E staining), 2 discontinuous slices per animal were quantified; all crypts observed on the slices were included. For degranulation analysis of immunofluorescence staining, 20 crypts of each genotype were quantified.

### *In Vivo* LPS Treatment

*Ep* and littermate control mice received LPS (Cat. code: tlrl-eblps, InvivoGen, diluted to 5 mg/ml in endotoxin-free water, 200 μg LPS/30 g body weight) or 200 μl endotoxin-free water (mock treatment) by mouth. Mice were sacrificed 1.5 h after treatment, and intestinal tissues were processed as described above. Serial sections were used for HE and immunofluorescence staining of lysozyme and procryptdin.

### *In Vitro* LPS Treatment

Intestinal crypts were isolated as described (17). Pelleted crypts were suspended with 2 ml iPIPES buffer (10 mM PIPES, 137 mM NaCl) and then treated with LPS (2 μg/ml) or endotoxin-free water (mock). At the designated time points after treatment, 5, 10, or 15 min, crypts and supernatant were collected by centrifugation at 4°C. For immunoblotting, crypt lysates were prepared as described above. Supernatant was mixed with 1% protease inhibitor cocktail before subjected to immunoblotting.

### OptiPrep Gradient Assay

Mouse small intestinal crypts were homogenized in 500 μl HB buffer (250 mM sucrose, 20 mM Tris-HCl, 1 mM EDTA, pH 7.4) with protease inhibitor cocktail followed by a centrifugation at 1,000 g for 10 min at 4°C to obtain post-nuclear supernatant. 50 μl of the supernatant was saved as input control, 400 μl of the supernatant was loaded onto 20 ml linear 5–50% linear Optiprep gradient (Axis-Shield, Norway) in HB buffer with a Gradient Master (Biocomp, USA) and centrifuged at 30,000 rpm for 16 h in an SW41 rotor (Beckman, USA) at 4°C. Thirteen equal fractions were collected from top to bottom and protein was precipitated by TCA. The precipitated protein and input control were analyzed by SDS-PAGE and immunoblotting.

### Statistics

All values in the text are shown as mean ± s.e.m. The two-way ANOVA test and Tukey test were used in the microbiota analysis. Student’s t-test was used in other experiments to compare means of two groups and Tukey test was used to compare means of more than two groups. The differences of *p <*0.05 were considered as statistically significant.

## Results

### Altered Composition in Fecal Microbiota of HPS-1 Patients and *ep* Mice

**Figure 1:**
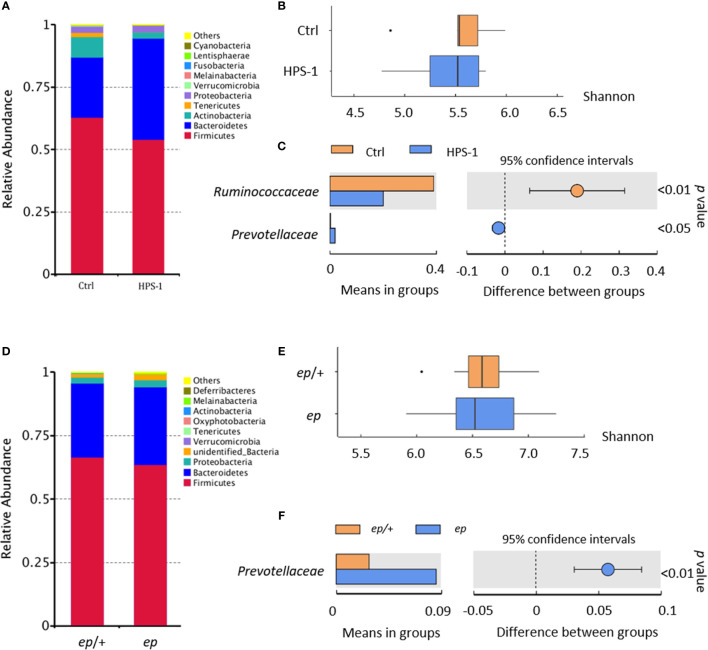
Fecal microbiota analysis reveals altered composition of HPS-1 patients and HPS-1 model mice. **(A)** According to the annotation results of species, the species with the largest abundance ranking in the top 10 in phylum level are shown in the histogram of relative abundance. **(B)** Shannon diversity of control and HPS-1. HPS-1 5.43 vs. control 5.55, *p > *0.05, not significant (N.S.) **(C)** The graph shows the abundance of dominant families (LDA score > 4) between groups and the confidence degree of inter-group differences. Bars in the graph represent the mean distribution value of the species with significant abundance difference between HPS-1 and control group. The leftmost endpoint of each circle in the figure represents the lower limit of the 95% confidence interval of the mean difference, and the rightmost endpoint of the circle represents the upper limit of the 95% confidence interval of the mean difference. The center of the circle represents the difference in the mean. The group represented by the color of the circle is the group with a high mean value. At the far right of the results is the *p*-value of the inter-group significance test for the specie of difference. *Prevotelleaceae* LDA score 4.94, abundance 27.56% in HPS-1 vs. 0.19% in control, *p < *0.05; *Ruminoccacceae* LDA score -5.04, abundance 20.00% in HPS-1 vs. 38.96% in control, *p < *0.001. **(D)** Relative abundance of adult WT and *ep* mice determined by 16S DNA sequencing and taxonomic analysis. *Firmicutes* 41.00% in WT vs. 30.65.68% in *ep*, *p < *0.05. **(E)** Shannon diversity of WT and *ep* mice fecal microbiome. *ep* 6.56 vs. WT 6.65, *p > *0.05, N.S.,*ep* mice microbiome diversity shows higher variation within the genotype group. Fecal samples were taken from gender and age paired adult mice littermates. **(F)** The abundance of dominant family between WT and *ep* mice and the confidence degree of inter-group differences. *Prevotellaceae* LDA score 4.52, abundance 2.81% in WT vs. 7.49% in *ep*, *p < *0.001. 8 mice of each genotype were included.

Fecal samples of 3 HPS-1 patients (age 6–7 years, **Table S1**) from 3 unrelated families and 4 healthy children (age 6–12 years) from 4 unrelated families along with samples from their parents were collected for 16S rDNA sequencing. Taxonomy-based analysis showed no significant gross alteration in the phylum composition of HPS-1 fecal microbiome compared to the control group (**Figure 1A**). Shannon diversity revealed no differences between HPS-1 patients and controls (**Figure 1B**). LDA effect size (LEfSe) is an analytical tool used to find and interpret the biomarkers (genes, pathways, classification units, etc.) between groups of high-dimensional data. It most likely explains differences between classes by coupling standard tests for statistical significance with additional tests encoding biological consistency and effect relevance, and emphasizes statistical significance and biological correlations (25). To identify the characteristics of different abundances and the associated categories, LEfSe was used to interpret the comparison of HPS-1 patients and controls. *Prevotellaceae* in the phylum Bateroidetes is the trait type in HPS-1 patients at the family level while healthy controls show predominantly *Ruminoccacceae*, a family in phylum Firmicutes (**Figure 1C**). Furthermore, no dominant species of the HPS-1 parent group were found in comparison to the healthy parent group (**Figure S1**). Similarly, a previous study reported that *Prevotella* is more abundant in fecal samples from patients with diarrhea-predominant irritable bowel syndrome compared to healthy controls (26). Thus, HPS-1 patients, but not their parents, had altered composition of fecal microbiota.

The HPS-1 mouse mutant, *ep* (or *ep/ep*), carries a spontaneous IAP element insertion in the *Hps1* gene resulting in the absence of HPS1 protein (27). To investigate whether *ep* mice have the risk of IBD as shown in HPS-1 patients, we sequenced fecal 16S rDNA from age and gender paired adult wild-type (WT) and *ep* mice. Taxonomy-based analysis suggested that the relative abundance of *Firmicutes* decreased in *ep* mice (**Figure 1D**). Shannon diversity analysis revealed no significant differences between *ep* mice and WT controls, but a higher individual variation within the *ep* mice group was observed (**Figure 1E**). LEfSe revealed that *Prevotellaceae* in phylum Bacteroidetes was also the dominant type in *ep* mice (**Figure 1F**). Taken together, these results suggest that HPS1 deficiency affects the intestinal microbiota both in humans and mice, which may confer a risk for IBD development.

### HPS1 Deficiency Leads to Altered LDCV Morphology in PCs

**Figure 2:**
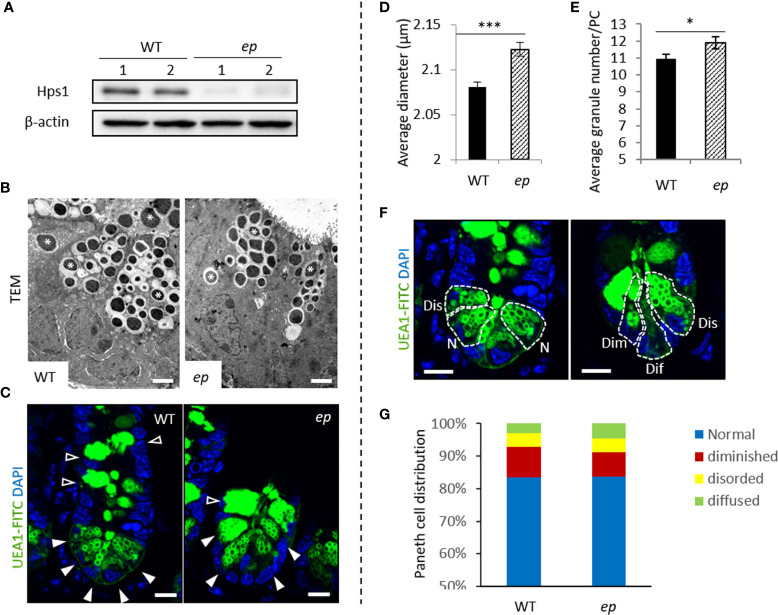
Morphology of Paneth cell LDCVs in unchallenged control and *ep* mice. **(A)** Hps1 expression in mouse small intestine crypts. WT mice express Hps1 while HPS-1 mutant *ep* mice lack Hps1 protein. **(B)** Representative transmission electron microscopy image of Paneth cell LDCVs in chemical fixed WT and *ep* mice ileal tissue. LDCVs were marked by *. Bar = 2 μm. **(C)** Representative images of FITC-UEA1 labeled Paneth cells and Goblet cells, Paneth cell LDCVs are shown by white arrow, Goblet cell granules are shown by empty arrow. Bar = 20 μm. **(D)** Quantification of the average LDCV diameter of *ep* Paneth cells and WT. WT 2.08 ± 0.006 μm, *ep* 2.12 ± 0.007 μm, ***p < 0.001. Data are presented as the mean ± s.e.m. **(E)** Quantification of the average LDCV numbers per Paneth cell. WT 10.95 ± 0.26 granules/cell, *ep* 11.88 ± 0.36 granules/cell, *p < 0.05. Data are presented as the mean ± s.e.m. of average LDCV number per Paneth cell with the indicated genotypes. **(F)** Paneth cell types by LDCV number and morphology. Normal (N) cells contain more than 10 UEA-1 labeled LDCVs at the top of the Paneth cells. Disordered (Dis) cells contain normal number of LDCV but some LDCVs occur at the basal end. Diminished (Dim) cells contain less than 10 labeled LDCVs and among which some enlarged granules were observed. Diffused (Dif) cells are UEA-1 positive but contain no typical LDCV structure. Bar = 20 μm. **(G)** Quantification of the percentage of each Paneth cell type according to the Paneth cell phenotypes in **(F)**, WT PCs 10% diffused, 4% disordered and 3% diminished; *ep* PCs 7% diffused, 4% disordered and 5% diminished. Data are presented as the average value of the percent of Paneth cells with the indicated phenotype out of the total number of Paneth cells counted for each mouse.

To determine whether HPS1 is involved in regulating the biological function of PCs, we first detected HPS1 expression in murine small intestine crypts containing PCs, transient amplifying cells and stem cells. Western blotting confirmed that HPS1 protein was expressed in WT crypts, and was absent in *ep* crypts (**Figure 2A**). Unfortunately, we did not test the intracellular localization of HPS1 because our HPS1 antibody did not work well for immunostaining.

We then analyzed the phenotypes of LDCVs and PCs in *ep* mice to investigate the role of HPS1 in PCs. *ep* PC LDCVs displayed a typical dense core structure compared to WT PC LDCVs (**Figure 2B**). We visualized PC LDCVs with FITC-UEA-1 which specifically binds to glycoprotein or fucose residue of lipopolysaccharide on the granule membrane (**Figure 2C**). PC morphology analysis was performed as described (6) (**Figure 2F**). For both *ep* and control mice, PCs with normal morphology were predominant (an average of 84% in WT mice vs. 83% in *ep* mice). The proportion of abnormal PC subtypes in *ep* mice had no significant changes compared to the WT mice (**Figure 2G**). Furthermore, quantification of the immunofluorescence staining images showed that there was a significantly enlarged average diameter of LDCVs in *ep* mice compared to WT (**Figure 2D**) and a significantly increased number of LDCVs per PC in *ep* mutants (**Figures 2E**, **S2**). Thus, HPS1 deficiency is associated with an abnormal morphology in PC LDCVs.

### No Apparent Changes of Lysozyme and Procryptdin Expression in *ep* PCs

**Figure 3:**
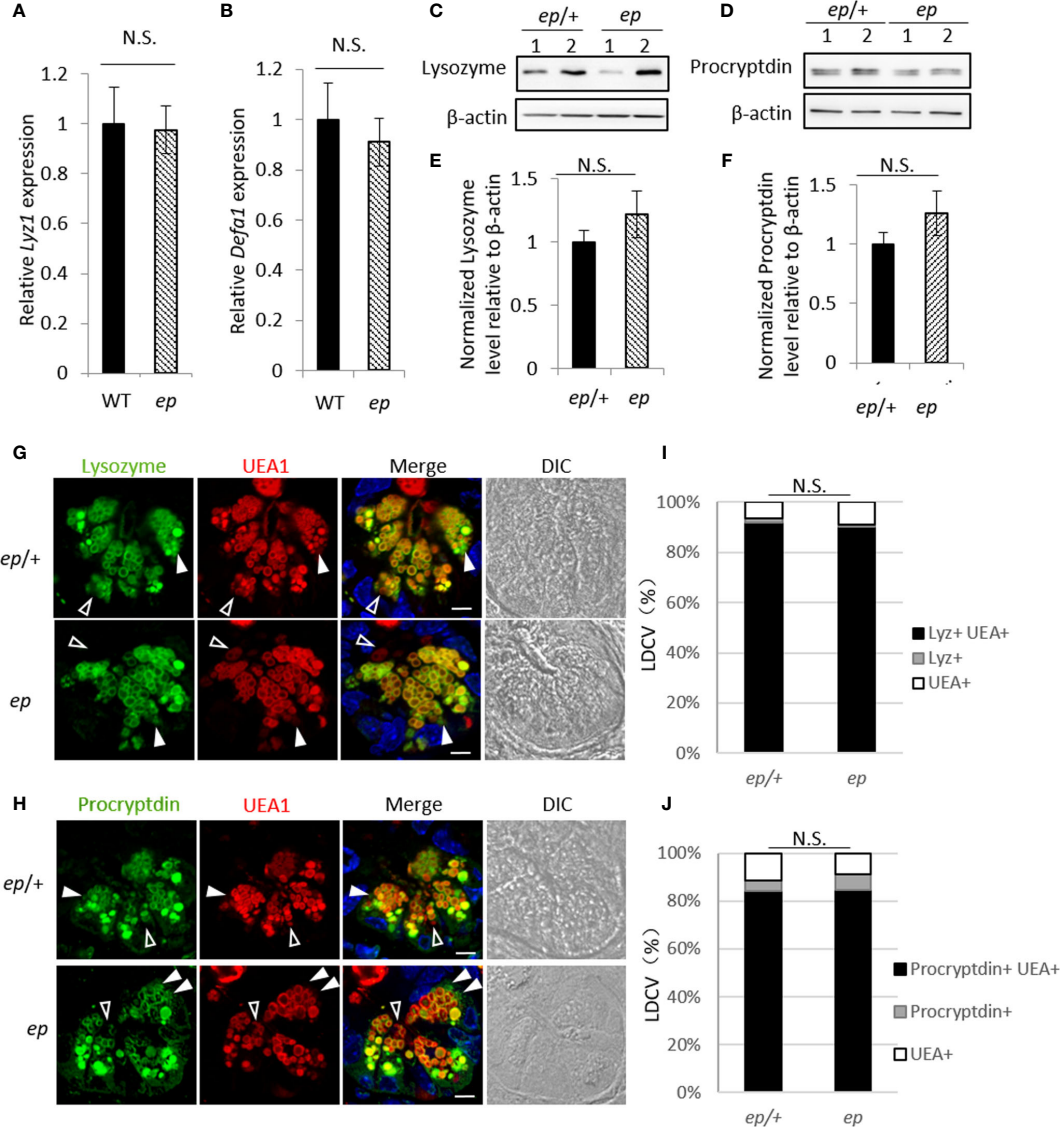
Expression and subcellular localization of Paneth cell anti-microbial peptides. **(A, B)** qRT-PCR analysis of *Lyz1* **(A)** and *Defa1* **(B)** showed no difference in the expression of lysozyme or procryptdin between *ep* and WT ileum (*Lyz1* 0.98, *p* > 0.05, N.S.; *Defa1* 0.91, *p* > 0.05, N.S.). **(C, D)** Representative immunoblots of lysozyme and procryptdin in *ep*/+ and *ep* ileum. **(E)** Quantification of lysozyme protein level in *ep* ileum shows no significant change compared to *ep*/+ (n = 5, *p* > 0.05, N.S.). **(F)** Quantification of procryptdin protein level in *ep* ileum shows no significant change compared to *ep*/+ (n = 5, *p* > 0.05). **(G, H)** Representative immunofluorescence images of *ep*/+ and *ep* ileum section stained with lysozyme and procryptdin. Paneth cell LDCVs were labeled by Rhod-UEA1 and nuclei were stained with DAPI. White arrows show granules lack of UEA1 signal, empty arrows show granules lack of lysozyme or procryptdin signal. Bar = 10 μm. **(I, J)** Percentage of each category described in (G, H), each category *p >*0.05, N.S. LDCVs from 20 PCs per mouse, 3 mice for each genotype were quantified.

To explore whether the abnormal LDCV morphology leads to altered expression of the contents in *ep* PCs, we carried out immunofluorescence staining of mouse small intestine slices. Lysozyme and procryptdins, major proteins of LDCVs which are main functional AMPs associated with mucosal anti-microbial activity and gastrointestinal innate immune response, were located within granule structures and were partially co-localized with Rhod-UEA1, which concentrates on the surface of mature LDCVs (28, 29), in both control heterozygotes (*ep*/+) and *ep* mice. Lysozyme and procryptdin colocalized with UEA1 at most LDCVs while a small amount of LDCVs were only positive for the antimicrobial peptides (white arrow) or only positive for UEA1 (empty arrow) (**Figures 3G, H**), but the distribution of each AMPs showed no difference between *ep* and heterozygotes (**Figures 3I, J**). We next tested whether the transcription and expression of the two proteins in *ep* PCs was changed. *Lyz1*, the gene encoding lysozyme, and *Defa1*, a representative gene of procryptdins, were quantified by real time RT-PCR. The expression levels of the two genes in the small intestine of *ep* mice were not significantly changed compared with WT (**Figures 3A, B**). Furthermore, no significant changes of lysozyme and procryptdins in the small intestine of *ep* mice were detected by Western blotting although the high variations were observed among samples (**Figures 3C–F**). These results suggest that HPS1 deficiency does not affect the transcription or expression of lysozyme and procryptdins.

### Lysozyme Secretion Is Impaired in *ep* PCs

**Figure 4:**
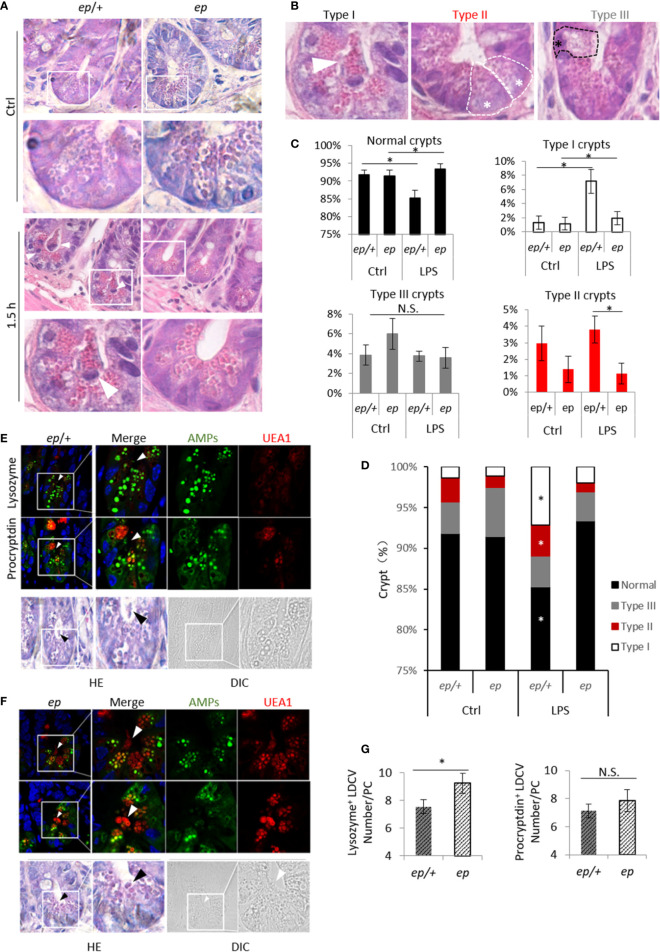
Paneth cells degranulation after LPS treatment. **(A)** HE staining of small intestine sections from untreated and LPS treated *ep/*+ and *ep* mice, mature Paneth cells featuring LDCVs labeled by eosin, white arrow heads show granules in lumen and light eosin staining Paneth cells undergo degranulation. **(B)** Small intestine crypts typed by Paneth cell morphology: type I crypt featuring granules in lumen (white arrow), type II crypt featuring mature Paneth cells with LDCVs and no significant degranulation in lumen (white *), type III crypt featuring Paneth cells with eosin negative vesicles and no significant degranulation in lumen (black *). **(C, D)** Quantification of crypt in **(A)** as described in **(B)** between control and LPS treated *ep*/+ and *ep* mice. *p < 0.05. Small intestine sections from 5 mice of each genotype per group were quantified. **(E, F)** Paneth cell LDCVs were visualized by eosin and Rhod-UEA1 (red) while AMPs were labeled by immunofluorescence (green). Black arrows indicate LDCVs that were secreted into crypt lumen. **(G)** Statistic analysis of procryptdin^+^ LDCV and lysozyme^+^ LDCV numbers in treated *ep/+* and *ep* Paneth cells. Procryptdin^+^ LDCV numbers in treated *ep/+* (7.13 ± 0.49 granules/cell, 5 mice, n = 150) and *ep* (7.85 ± 0.78 granules/cell, 5 mice, n = 135) Paneth cells showed no difference (*p* > 0.05, N.S.) while *ep* Paneth cells contain more lysozyme^+^ LDCVs (7.55 ± 0.51granules/cell, 5 mice, cell number = 120) than *ep* (9.24 ± 0.73 granules/cell, 5 mice, cell number = 108, *p < 0.05).

To investigate whether the abnormal LDCV morphology in *ep* mice has an effect on PC function, age and gender matched *ep*/+ and *ep* mice were orally treated with lipopolysaccharide (LPS), which is a main component of gram negative bacterium cell wall and a TLR2/4/9 agonist triggering PC degranulation and secretion of AMPs (30). Hematoxylin-eosin (HE) staining analysis of small intestine sections harvested after 1.5 h revealed PC degranulation in control *ep*/+ mice while the *ep* PCs with mature morphology had no obvious degranulation (**Figure 4A**). We classified intestinal crypts according to the morphology and distribution of LDCVs in PCs (31, 32). Type I and type II crypts contain empty PCs or secreted LDCVs in crypts lumen, type III crypts contain non-degranulated PCs with abnormal vacuolar structures (**Figure 4B**). Statistical analysis showed that after 1.5 h of LPS treatment, the proportion of type I and type II crypts in *ep*/+ small intestine sections was significantly increased compared to equally treated *ep* mice and untreated *ep*/+ mice (ctrl), while the proportion of type III crypts that were not supposed to be affected by LPS treatment showed no significant differences between the two genotypes (**Figures 4C, D**). We observed that PCs with type I and II crypts in treated *ep*/+ mice had fewer lysozyme positive granules and UEA1 labeled LDCVs, but the number of procryptdin positive granules between the two genotypes showed no significant difference (**Figures 4E–G**).

**Figure 5:**
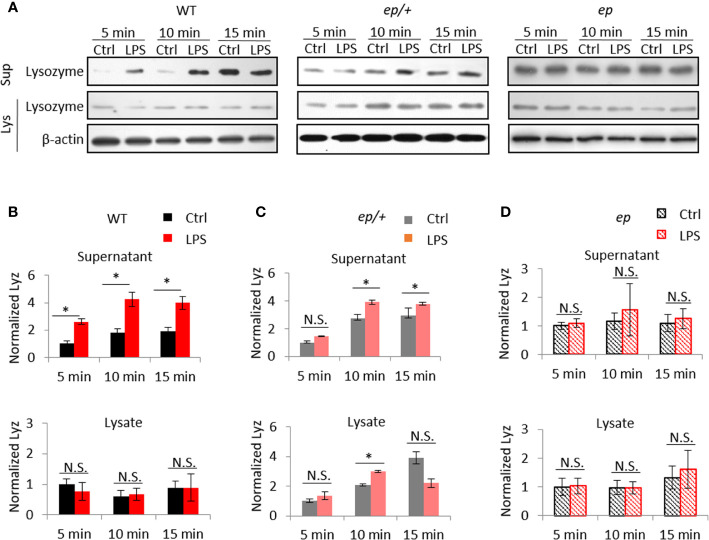
Lysozyme secretion defects in *ep* mice crypts. **(A)** Representative immunoblots for lysozyme in supernatant (Sup) and lysate (Lys) from isolated crypts stimulated with 2 μg/ml LPS for 5, 10, 15 min. **(B)** Lysozyme in WT supernatant increased after 5 min stimulation (2.61 ± 0.22, 4 mice, *p < 0.05) and remained till 10 min (2.36 ± 0.53, 4 mice, *p < 0.05) while lysozyme in WT crypts lysate remained unchanged (*p* > 0.05, N.S.). **(C)** Lysozyme secretion of *ep/+* crypts showed a delayed (5 min, *p* > 0.05, N.S.) and mild (10 min 1.41 ± 0.03, *p < 0.05, 15 min 1.28 ± 0.12, *p < 0.05, 4 mice) increase after LPS stimuli, while lysozyme in *ep/+* crypts lysate showed an increase after 10 min stimulation (1.44 ± 0.08, *p < 0.05) and recovered at 15 min (0.56 ± 0.31, *p* > 0.05, N.S.). **(D)** Lysozyme in *ep* crypts supernatant remain unchanged after LPS stimuli (5 min 1.09 ± 0.15, *p* > 0.05, 10 min 1.34 ± 0.91, *p* > 0.05, 15 min 1.14 ± 0.35, *p* > 0.05, N.S.), and lysozyme in *ep* crypts lysate remain unchanged after LPS stimuli (5 min 1.03 ± 0.27, *p* > 0.05, 10 min 0.99 ± 0.20, *p* > 0.05, 15 min 1.21 ± 0.66, *p* > 0.05, N.S.). Four mice per group were quantified. Quantification of lysozyme in (B–D) is normalized by loading control β-actin.

Lysozyme secretion after LPS treatment was analyzed with isolated small intestinal crypts *in vitro*. We treated isolated crypts with LPS and followed lysozyme secretion into the supernatants. The results showed that lysozyme in supernatant fraction from isolated WT crypts was significantly increased 5 min after LPS treatment and reached a peak at 10 min, while un-secreted lysozyme in lysate fraction remained unchanged (**Figures 5A, B**). In control *ep*/+ mice, supernatant lysozyme had similar changes to WT, while intracellular lysozyme content increased slightly at 10 min of LPS treatment compared to the mock treatment but decreased at 15 min (**Figure 5C**). However, lysozyme in either supernatant or lysate fractions from *ep* crypts did not show such changes after LPS treatment compared to mock treatment (**Figure 5D**). Taken together, HPS1 deficient PCs were unable to release lysozyme in response to LPS stimulation, and heterozygous *ep*/+ mice showed a slight defect in lysozyme secretion possibly due to dose effects of HPS1 protein.

### HPS1 Deficiency Affects the Subcellular Localization of VAMP7

To further study the underlying mechanism of impaired lysozyme secretion in *ep* PC LDCVs, inferred from the function of HPS1 in melanosomes (19), we reasoned that HPS1 may participate in the recycling of v-SNARE proteins from PC LDCVs, which may be important for the maturation of LDCVs and concomitantly the release of LDCV cargo. HPS1 has been implicated in the recycling of VAMP7 from melanosomes to REs through the activation of Rab38/Rab32 (19). To address whether this pathway functions in PC LDCVs, we measured the total protein level and subcellular location of VAMP7 in ileum. Western blotting was performed to determine the expression of Rab38, Rab32, VAMP7, and other SNARE or regulatory proteins in small intestine tissue (**Figures S3 A–C**). The protein levels of VAMP7, Rab38, and Rab32 (**Figure S3 D–F**) together with VAMP4, VAMP8, synaptotagmin, and snapin (**Figures S3G, H**) were not affected by either the loss of HPS1 or upon LPS stimuli.

**Figure 6:**
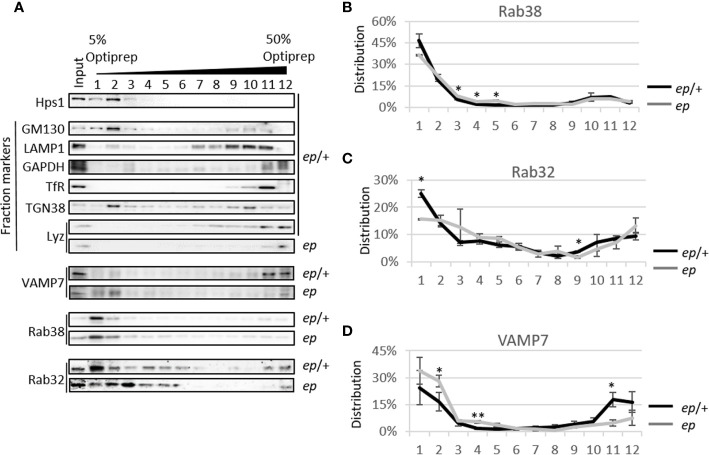
Protein level and subcellular content analysis of v-SNARE and Rab proteins in mice crypts. **(A)** Representative image of 5~50% Optiprep gradient of *ep/+* and *ep* mice small intestine crypts. VAMP7 mainly appears at the 11th and 12th fractions labeled by lysozyme and endosomal component TfR and a second peak appears at the first two fractions labeled by GM130 and TGN38, while it increases in the 4th fraction in *ep* crypts. Rab38 mainly appears at the first two fractions of both genotypes while its concentration increases in the middle fragments in *ep* crypts. Rab32 has similar distributional pattern as Rab38 but more widely distributed in 3rd~6th fractions, but less in the 11th fraction labeled by TfR in *ep* crypts. **(B)** Statistical analysis of Rab38 distribution in **(A)**. In *ep* crypt, the increase of Rab38 in the middle density fragments is significant (the 3rd~5th fractions, *ep/+* crypts 9%, *ep* crypts 16%, *p < 0.05). **(C)** Statistical analysis of Rab32 distribution in **(D)**. Rab32 in *ep* crypts significantly decreases at the low density fragment (the 1st fraction: *ep/+* crypts 25%, *ep* crypts 16%, *p < 0.05) and at the 9th fraction (ctrl 4%, *ep* 2%, *p < 0.05). **(D)** Statistical analysis of VAMP7 distribution in **(A)**. VAMP7 in *ep* crypts significantly increases in TGN38 and GM130 enriched fraction (the 2nd fraction, ctrl crypts 17%, *ep* crypts 28%, *p < 0.05) and the 4th fraction (*ep/+* crypts 2%, *ep* crypts 6%, **p < 0.01), and decreases in the RE enriched 11th fraction (*ep/+* crypts 18%, *ep* crypts 5%, *p < 0.05). 4 mice of each genotype were analyzed, values are means ± s.e.m. of three replicates of immunoblotting data.

**Figure 7:**
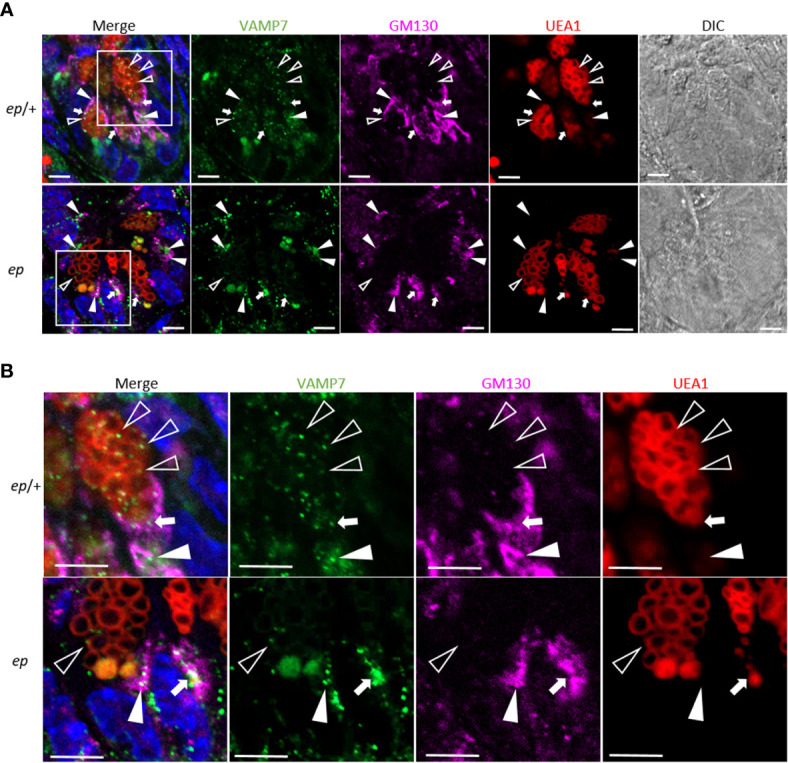
Altered subcellular location of VAMP7 in *ep* Paneth cells. **(A)** Representative immunofluorescence images of *ep*/+ (Ctrl) and *ep* ileum section stained with anti-VAMP7 (green) and anti-GM130 (purple). UEA1 (red) indicates LDCVs. The arrow heads indicate the co-localization of VAMP7 and GM130, the open triangles indicate the co-localization of VAMP7 and UEA-1 and the white arrows indicate the co-localization of all those three makers. **(B)** Boxed areas are shown at higher magnification. Bar = 5 μm.

To explore whether the subcellular distribution of these proteins is altered, lysates of small intestinal crypts from *ep* mice and littermate *ep*/+ controls were fractionated by gradient centrifugation (**Figure 6A**). Judging by the organelle markers, Golgi (GM130) and TGN (TGN38) were enriched in fraction 2, lysosomes (LAMP1) in fractions 9–11, endosomal structures (33) in fraction 11, and LDCVs (Lyz) in fraction 12. Among these proteins, VAMP7 in *ep*/+ crypts concentrated at the high density fractions labeled by LDCV cargo lysozyme (the 11th fraction 18%) and endosomal component TfR (the 12th fraction, 16%) and at the low density fractions enriched with Golgi and TGN (the 2nd fraction, 17%). However, in *ep* crypts, VAMP7 partially shifted from the 11th fraction (5%) to the 2nd fraction (28%) (**Figure 6D**). This distributional shift suggests that in the absence of HPS1, VAMP7 failed to be removed from LDCVs and mostly remained in the Golgi and TGN (**Figure 7**). We also observed a slight shift of Rab38 to the middle density fractions (the 3rd to 5th fractions) in *ep* crypts (**Figure 6B**), and Rab32 had similar distributional shift in *ep* crypts (**Figure 6C**). These results suggest that deficiency in HPS1 GEF activity alters the membrane association of Rab32/38 which may affect the removal of VAMP7 from LDCVs, and that loss of HPS1 does not affect the cargo sorting-in process but rather compromises the pathway for cargo sorting-out, thus impairing the maturation of LDCVs.

## Discussion

In this study, we reported the abnormal morphology (increased number and enlarged size) in HPS1 deficient LDCVs, a type of LROs in Paneth cells. This alteration may impair the secretion of lysozyme, a key anti-microbial peptide associated with mucosal anti-microbial activity and gastrointestinal innate immune response. The abnormal secretion of lysozyme thereafter leads to the changes in the composition of intestinal microbiota, which may confer a risk for IBD development. Mechanistically, HPS1 is likely associated with the recycling of VAMP7 from LDCVs to promote the maturation of LDCVs. The immature LDCVs are defective in lysozyme secretion. This mechanism is conserved in melanosome maturation (19). Exploring the role of HPS1 in the biogenesis of LDCVs in Paneth cells provides new insights into the susceptibility of HPS-associated IBD and the role of HPS1 in innate immunity.

HPS1 functions as a GEF of Rab32/38 that mediates the conversion of Rab32/38 between the inactive GDP binding form and the active GTP binding form (20). In the melanocyte cell line MNT-1, Rab38 locates at melanosomes and indirectly interacts with VAMP7 *via* VARP (34). In this study we reported that in normal Paneth cells, Rab38, Rab32, and VAMP7 partially co-localized at LDCVs. VAMP7 is likely to be routed back to RE after recycling from LDCVs. In HPS1-deficient Paneth cells, VAMP7 shifted from LDCVs and endosome-associated fractions to the Golgi fractions, suggesting that the HPS1-Rab32/38-VAMP7 axis is likely to be a general mechanism in the biogenesis of LROs. This raises the possibility that LDCVs and REs interact for VAMP7 recycling by the action of HPS1. However, the machinery of how Rab32/38 mediates VAMP7 recycling awaits further investigation.

The NOD2-LRRK2-Rab2a pathway is an important molecular switch in the maturation of PC LDCVs that specifically regulates the sorting of lysozyme (17). LRRK2 protein contains a leucine rich repeat domain, an armadillo repeat, a kinase domain, an anchor protein domain, a RAS (GTPase) domain and a WD40 repeat (35). *LRRK2* is also associated with Parkinson’s disease (36) and is involved in vesicle transport, autophagy, cytoskeleton formation and mitochondrial function (37). The transportation and kinase activity of cytoplasmic LRRK2 are regulated by Rab32/38. LRRK2 binds to Rab32/38 in a GTP-dependent way *via* the armadillo repeat domain, and the complex is mainly colocalized at the endosomes and trafficking vesicles (38). Overexpression of constitutively activated mutant Rab32 led to increased LRRK2 localization to late endosomes and multi-vesicular bodies (39). We observed a slight shift of Rab2a in our gradient fractionation assay (data not shown). Whether HPS1 is the GEF of Rab2a, or regulates the NOD2-LRRK2-Rab2a pathway *via* its interaction with Rab32/38 needs to be investigated.

Case reports have shown that among eleven HPS subtypes the frequency of HPS-1 is much higher than the others (18), and about 10% of the reported HPS-1 cases had colitis or CD-like IBD (3). Development of HPS1-IBD, although it is at low frequency, may depend on complex genetic and environmental factors. There were no indications to perform endoscopy for our HPS-1 patients due to their young ages and no IBD symptoms. CD-like IBD have also been reported in some cases with HPS-4 or HPS-6 (3, 4). HPS1 and HPS4 form a tight BLOC-3 complex which functions as a GEF for Rab32/38 (20). BLOC-3 and Rab32/38 are involved in cargo removal (e.g. VAMP7) during LRO maturation (19). HPS6, HPS5, and HPS3 form the BLOC-2 complex which is known to be involved in cargo sorting during LRO biogenesis. BLOC-1 and AP-3 cooperate in this cargo sorting pathway (1, 40). During LRO biogenesis, the HOPS complex plays an important role in autophagy which is required for LRO maturation probably by degradation of excessive membrane or proteins (41). Thus, deficiency in any of these HPACs may have defects in LRO biogenesis, which thereafter impairs LRO secretion. Regarding this general mechanism of LRO dysfunction in HPS patients, it is plausible to have multi-organelle defects of tissue-specific LROs such as melanosomes in melanocytes and LDCVs in PCs. However, symptoms or severity of HPS-associated IBD may vary in different HPS subtypes or in different susceptibility conditions of HPS patients.

The secretion of lysozyme and the release of LDCV are essential for the maintenance of enteral homeostasis. We here report the altered fecal microbiota composition in HPS1-deficient *ep* mice similar to the CD mouse models (42) and CD patients (43), reflecting a vulnerable enteral homeostasis. The interaction of genetics and the environment contributes to CD pathogenesis. Because the mutant *ep* mice were bred under specific pathogen-free facilities, *ep* did not have typical symptoms of IBD. HPS-1 patients in this study also had no CD symptoms at their young ages, but the fecal microbiota dominant phenotype *Prevotellaceae* including *Prevotella* predisposes a higher risk of diarrhea-predominant irritable bowel syndrome (26). However, whether fecal microbiota transplantation can alleviate the development or symptoms of HPS-associated IBD requires further investigation.

## Data Availability Statement

The raw data supporting the conclusions of this article will be made available by the authors, without undue reservation.

## Ethics Statement

The studies involving human participants were reviewed and approved by the internal review board of the bioethics committees of Beijing Tongren Hospital, Capital Medical University. Written informed consent to participate in this study was provided by the participants’ legal guardian/next of kin. The animal study was reviewed and approved by Institutional Animal Care and Use Committee of Institute of Genetics and Developmental Biology, Chinese Academy of Sciences.

## Author Contributions

WL, ZL, and JY designed the research and wrote the manuscript. WL and ZL supervised the study. XH and CH analyzed the data and provided funding support. JY led the experimental investigation. QZ, YP, ZH, LY, YY, ZZ, and CZ performed some assays and analyzed the data. AW and TL diagnosed and collected the HPS patient samples. All authors contributed to the article and approved the submitted version.

## Conflict of Interest

The authors declare that the research was conducted in the absence of any commercial or financial relationships that could be construed as a potential conflict of interest.

## Abbreviations

AMP, anti-microbial peptide; BLOC, biogenesis of lysosome-related organelles complex; CD, Crohn’s disease; *ep*, pale ear mutant mouse; GEF, GDP/GTP exchange factor; HE, Hemotoxylin-Eosin staining; HPS, Hermansky-Pudlak syndrome; HPS1, Hermansky-Pudlak syndrome protein 1; IBD, inflammatory bowel disease; LDCV, large dense core vesicle; LPS, lipopolysaccharide; LRO, lysosome-related organelle; PC, Paneth cell; RE, recycling endosome; VAMP, vesicle-associated membrane protein; WT, wild-type.
