# Supplementary material for: HPS1 Regulates the Maturation of Large Dense Core Vesicles and Lysozyme Secretion in Paneth Cells
Source: Front Immunol. 2020 Nov 5;11:560110. doi: 10.3389/fimmu.2020.560110 (PMC7674556; doi:10.3389/fimmu.2020.560110)
Supplement: Supplementary file 1 [file DataSheet_1.pdf]

Table S1. Genotypes and clinical features of the HPS-1 patients in this study.

				Mutation alleles			Clinical manifestations							
Case ID	Gender	Age	Platelet dense granules	Paternal	Maternal	Platelet Western	Skin color	Hair color at birth	Iris color	Nystagmus	Hypopsia	Clinical dignosis	Colonoscopy	Colitis
H006	M	7 yr	Absent	c.398+5G>A	c.972delC	N/A	White	Brownish yellow	Brown	Yes	Yes	OCA2	N/A	No
H014	M	6 yr	Absent	c.1936_1937TA>CT	c.1932delC	Less HPS1	Normal	Brownish black	Blue gray	Yes	Yes	OA	N/A	No
H015	M	7 yr	N/A	c.533delA	c.81delG	N/A	White	Golden	Blue gray	Yes	Yes	OCA2	N/A	No

Variant nomenclature follows the HGVS guidelines (http://varnomen.hgvs.org/). The NCBI RefSeq transcripts used in this study is *HPS1* (NM_000195).

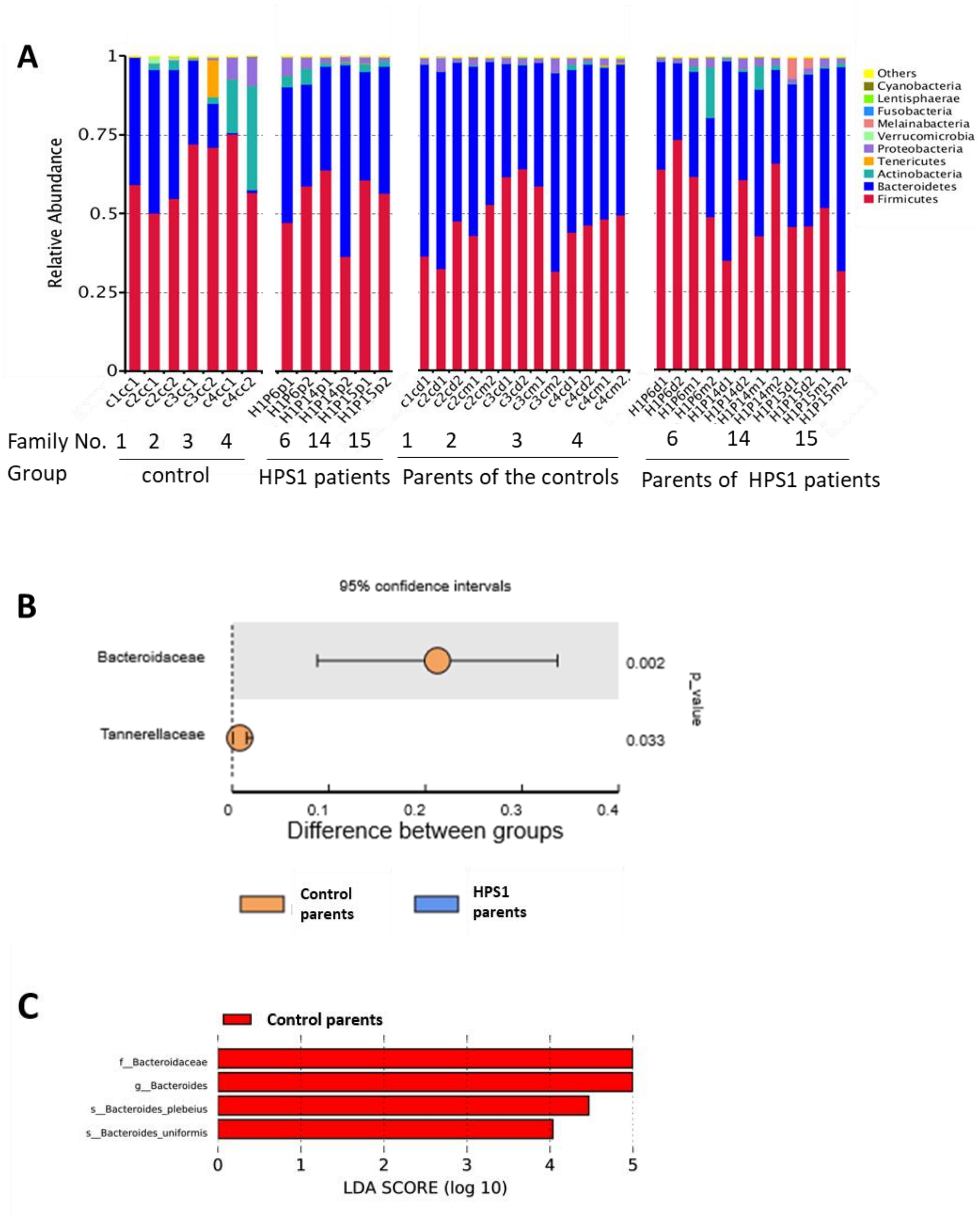

Supplemental Figure 1. Fecal microbiota analysis of HPS-1 patients.

(A) According to the annotation results of species, the species with the largest abundance ranking top 10 in phylum level are shown in the histogram of relative abundance. **(B)** The graph on the left shows the abundance of different species between groups. Bars in the

graph represents the mean value of the specie with significant abundance difference between HPS-1 parents group and control parents group. The figure on the right shows the confidence degree of inter-group differences. The leftmost endpoint of each circle in the figure represents the lower limit of the 95% confidence interval of the mean difference, and the rightmost endpoint of the circle represents the upper limit of the 95% confidence interval of the mean difference. The center of the circle represents the difference in the mean. The group represented by the color of the circle is the group with a high mean value. At the far right of the results is the *p*-value of the inter-group significance test for the specie of difference. **(C)** The histogram of LDA value distribution showed that the LDA score was greater than the set value (default was set to 4), that is, the biomarker with statistical difference between control parents and HPS-1 parents group. The length of the bar chart represents the influence of the different species.

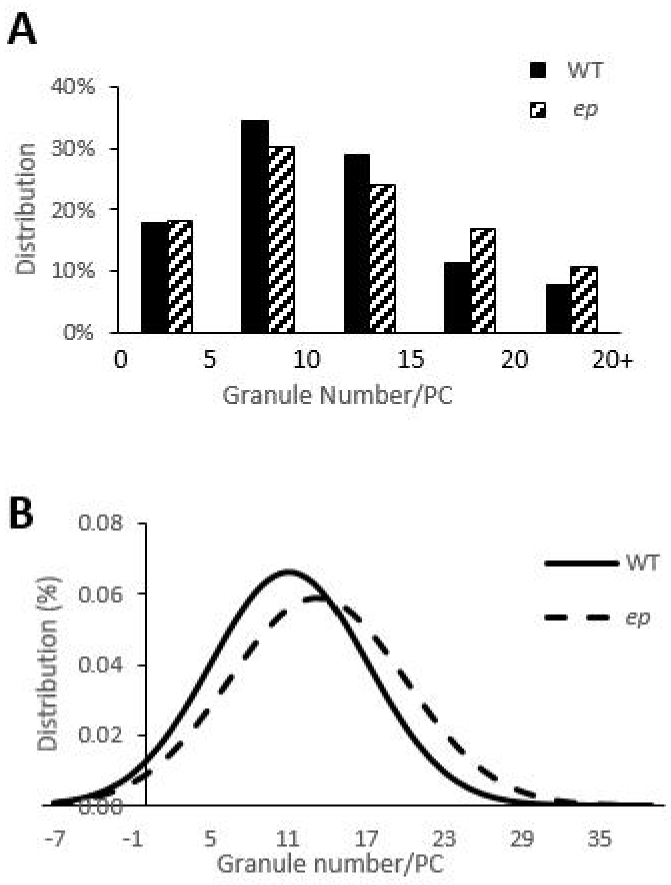

Supplemental Figure 2. Fecal microbiota analysis of HPS-1 patients.

(A) LDCV numbers per Paneth cell are presented as a frequency histogram. Data are presented as the percent of Paneth cells containing indicated number of LDCVs. More Paneth cells that contain more than 15 granules are observed in *ep* mice. **(B)** Normal distribution fitting curve of (A).

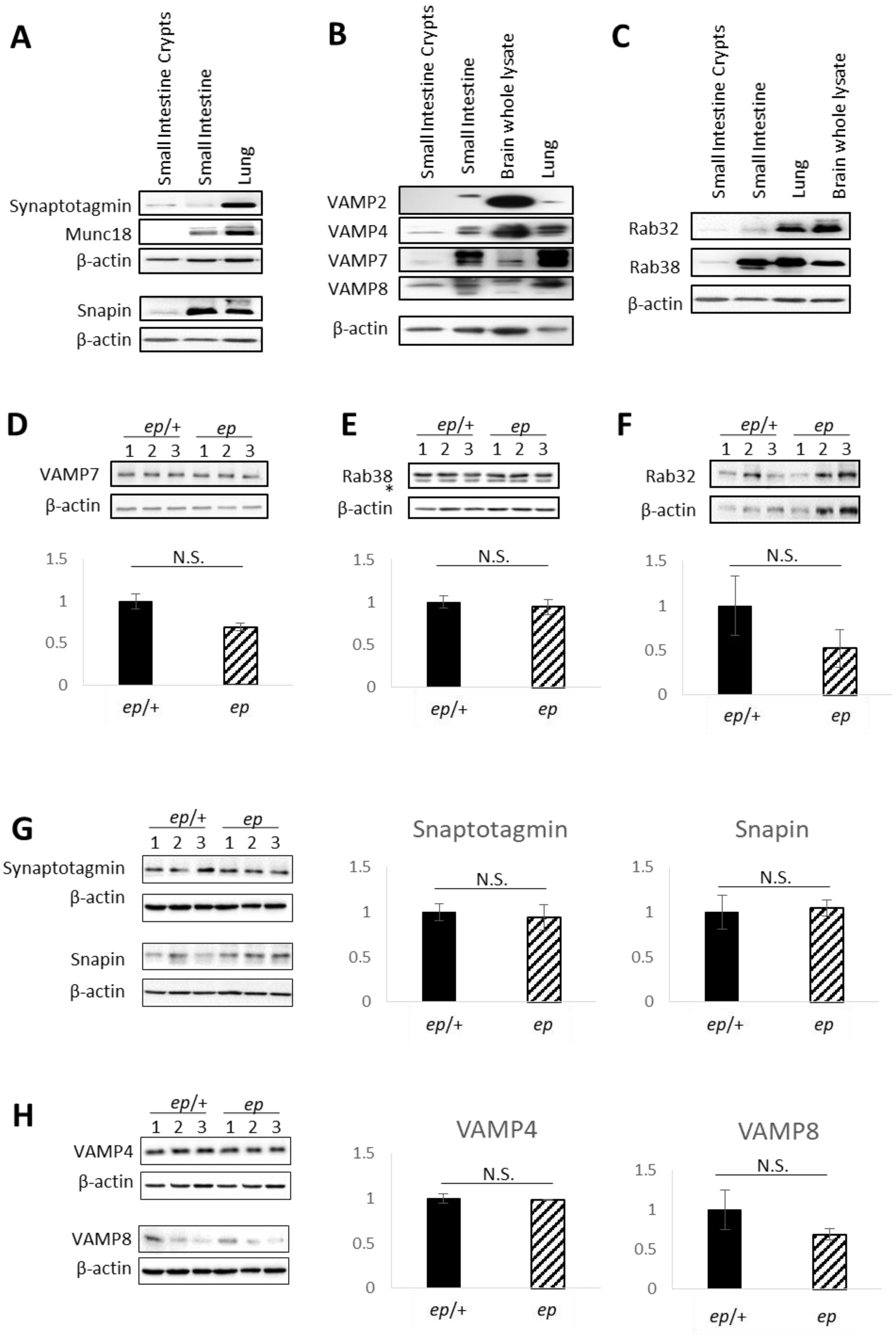
A
Small Intestine Crypts
Small Intestine
Lung
Synaptotagmin
Munc18
β-actin
Snapin
β-actin
B
Small Intestine Crypts
Small Intestine
Brain whole lysate
Lung
VAMP2
VAMP4
VAMP7
VAMP8
β-actin
C
Small Intestine Crypts
Small Intestine
Lung
Brain whole lysate
Rab32
Rab38
β-actin
D
ep/+
ep
1 2 3 1 2 3
VAMP7
β-actin
N.S.
1.5
1
0.5
0
ep/+
ep
E
ep/+
ep
1 2 3 1 2 3
Rab38
*
β-actin
N.S.
1.5
1
0.5
0
ep/+
ep
F
ep/+
ep
1 2 3 1 2 3
Rab32
β-actin
N.S.
1.5
1
0.5
0
ep/+
ep
G
ep/+
ep
1 2 3 1 2 3
Synaptotagmin
β-actin
Snapin
β-actin
Snaptotagmin
N.S.
1.5
1
0.5
0
ep/+
ep
Snapin
N.S.
1.5
1
0.5
0
ep/+
ep
H
ep/+
ep
1 2 3 1 2 3
VAMP4
β-actin
VAMP8
β-actin
VAMP4
N.S.
1.5
1
0.5
0
ep/+
ep
VAMP8
N.S.
1.5
1
0.5
0
ep/+
ep

Supplemental Figure 3. Expression of SNARE associate proteins in mice small intestine.

(A) In WT mice, synaptotagmin and snapin are expressed in small intestine crypts and are highly expressed in lung. No Munc18 expression was detected by immunoblotting in lysates of mouse small intestine crypts. **(B)** In WT mice, VAMP2 is highly expressed in brain and no VAMP2 expression was detected by immunoblotting in lysates of small intestine crypts. VAMP4, VANP7 and VAMP8 are expressed in small intestine crypts, small intestine, brain and lung of WT mice. **(C)** Expression of Rab32 and Rab38 in small intestine crypts, small intestine, lung and brain of WT mice. **(D-F)** Representative western blots and bar graphs of VAMP7, Rab38 and Rab32 in *ep/*+ and *ep* mice small intestines. Bar graph shows normalized protein level compared to untreated ep/+ mice, no significant change was seen between genotypes ($P > 0.05$, 7 mice were analyzed in each group, values are means ± s.e.m. of three replicates of immunoblotting data). **(G-H)** Representative western blot images and bar graphs of synaptotagmin, snapin VAMP4 and VAMP8, in small intestine tissues of *ep*/+ and *ep* mice. Bar graphs show normalized protein level relative to *ep*/+. For all statistics here, $P > 0.05$, N.S. 7 mice were analyzed in each group, values are means ± s.e.m. of three replicates of immunoblotting data.
